# The Druggists General Receipt Book, with Veterinary Formulary

**Published:** 1872-01

**Authors:** 


*The Druggists' General Receipt Book, with Veterinary Formulary.* By Henry Beasley. Philadelphia, Lindsay & Blakiston, 1871.

This book contains a receipt for making nearly everything made by druggists, and we should think that for them it would be valuable. The veterinary formulary must also be a very valuable guide to veterinary surgeons. It is not without attractions to physicians, though it is designed especially for druggists.

